# Doctors and the Peerage

**Published:** 1891-01-10

**Authors:** 


### The Hospital

A Weekly Journal of

Science, Medicine, Nursing, and Philanthropy.

*For Hospitals, Asylums, Infirmaries, Dispensaries, Medical Practitioners, Students, Nurses, and the Charitable Public.*

Vol. IX.—No. 224.] [January 10, 1891.

### DOCTORS AND THE PEERAGE.

A curious doctor wants to know if it is the fact that no medical man in this country has ever been raised to the Peerage; and if it be, why it is? So far as we are informed on the subject, there never has been a medical peer in England, and we have reason to believe that our information is quite correct. This is a very familiar, but also a very peculiar fact, and is worth inquiring into. A comparatively old civilisation like our own presents many curious and interesting features. The fact, for example, that a great many brewers sit on the pinnacles of the temple of State honours, while very few medical men get beyond the steps leading up to the front door, and none are permitted to advance further than the inner porch, is decidedly a curiosity. It is advantageous sometimes to dig down to the root of a thing; an examination of that often throws light upon the nature and development of the stem and branches and fruit. The root of medicine—at least, of scientific medicine—does not descend very deeply into the soil in this country. In the time of Elizabeth there was a great queen; and there were also great statesmen, great poets, great lawyers, and great priests: but where were the great doctors? It was not that there were no men of science in Elizabeth's time, for Bacon lived in that period, and in that period poured the vials of his contempt upon medicine. It is probable that he knew nothing of Harvey's great discovery of the circulation of the blood, although that discovery was made and announced, at least to the College of Physicians, some ten years before Bacon's comparatively early death. But even if he had known of Harvey's discovery, he would very likely have said that it ought to have been made sixteen hundred and sixteen years before the commencement of the Christian era rather than sixteen hundred and sixteen years after it, and that it would have been so made if physicians had been worth their salt. Such an opinion would be difficult to controvert.

If all medicine had begun to be scientific with Harvey's discovery of the circulation of the blood in 1616, there can be no doubt that by this time medical men would have taken their rightful position in the estimation of those who have the bestowal of State honours; and instead of being placed a long way behind rich brewers, contractors, and city merchants, they would have been advanced to a position a long way ahead of them. But Harvey's great effort seems to have exhausted the originality of the profession, not only for a generation, but for a century and more. Moreover, Harvey's discovery was not a discovery in practical medicine, as the common world understands practical medicine, but in physiology; and, as a matter of historical fact, the immediate improvement it effected in actual medical practice was almost or altogether *nil*. It is a curious and striking thing, too, that, even down to the time of the Hunters, the advance in intelligence and rational capacity among the great body of medical practitioners had been exceedingly small. If we except the discovery of Jenner, we may say that medicine at the beginning of the nineteenth century stood almost exactly where it did at the beginning of the eighteenth. When, therefore, we take even a moderately wide survey of British medicine and its history, we cannot but see that, compared with other branches of intellectual activity, its record is exceedingly brief. Where were the doctors when Chaucer was writing his "Canterbury Tales"; or, to go much further back, when Gregory, at the end of the sixth century, planned, and Augustine undertook, the conversion of England to the Catholic faith? Those medical men who are surprised and humiliated by the failure of the leaders of the State to understand and rightly appreciate the profound learning and rare intellectual capacity of some of the distinguished physicians and surgeons of the present day, forget that a dozen centuries before scientific medicine began to exist, priests and philosophers took all knowledge for their province, and brought to bear upon their work the acutest of intellects and the most elevated of characters. It is forgotten, too, that for centuries not only was medicine unscientific, but it was almost universally a mere pretence of knowledge and skill; and no man or body of men can live in ignorance which makes a show of knowledge without certain degradation and mental and moral collapse.

When all these facts are borne in mind, it is not greatly surprising that medicine and medical men should for so long a period have occupied a relatively inferior position in this country. Although nothing can in itself be more honourable than the healing art, yet men do not honour other men because of the titles which they assume or the professions which they follow, but for the dignity of their character and the practical utility and success of their work. Let any scientific medical man of the present day picture to himself what must have taken place five hundred years ago in nine out of every ten homes when the doctor was called in to deal with such serious diseases as peritonitis, typhoid fever, strangulated hernia, perforation of stomach or bowel, and the like; or when tracheotomy, ovariotomy, or other such operation needed to be performed. The poorest villager can command competent treatment under all these circumstances now. But at the period when the Barons at Runnymede were compelling John to sign the Great Charter, neither the king, nor the primate, nor the chief minister of State, nor any other person, whatever his dignity, could rely on medical services such as a third year's student might perform to-day with competent skill and assured success. There is, therefore, a very long and a very unfortunate historical excuse for the extremely cavalier manner adopted by the State towards the distinguished medical scientists of our own period.

But the time has fully come for a changed estimate of the public and imperial value of medicine and medical men. Physicians and surgeons have gradually worked their way, during the present century, from the rear-guard of the scientific army to the front ranks. It is probable that if any twenty of the leading scientists of the time were taken at random ten of them would be found to be doctors. Moreover, in the present day, whatever may have been the case in the past, science is one of the very chiefest of all the instruments of human advancement; and among all the sciences medicine, with her auxiliaries, physiology, chemistry, and public and private hygiene, is one of the most immediately and practically serviceable to all sorts and conditions of men. Will any intelligent person venture to deny this? If, therefore, medical men may not claim worthier recognition on account of their wealth, such recognition should clearly be offered to them for the magnitude and importance of the services which they render to all classes of persons in the State. Will any thoughtful man affirm that he who merely accumulates wealth for himself and his own family should be as generously rewarded by the public as he who ministers to both the body and the mind of the public, and who increases the health and vigour of both? It is to the honour of medical men that they seldom get rich. If they did they would certainly receive much more generous recognition at Imperial hands. We are quite sure that both the present Prime Minister and the last are well able to appreciate the value to the State of wide scientific acquirements, especially when those acquirements tend to clear and strengthen the mental vision as well as to promote public and private health. Those eminent and distinguished persons owe it to themselves to give the palm to him who best serves the public well-being, and not to him who clamours most loudly for it. A doctor, it is said, is well content with a baronetcy; and we hope this is so, at any rate in the case of that newest baronet and oldest physician, Sir Richard Quain. It is not, however, a question of what the doctors are content with, but of what the State owes to itself in its distribution of public honours. There are several British physicians and surgeons at the present time who, in personal dignity, scientific and liberal culture, and elevation of character, would adorn any rank, however exalted, to which they might be raised. It is not for such men as these to clamour for social dignities; and they never do so. They know that the work they have done and the benefits they have conferred upon the country are incomparably greater claims to honour and regard than any titles which the State could bestow. But whilst they are too self-respecting to ask, the State should not be too blind to give, or at least to offer. If brewers, and lawyers, and soldiers, and rich merchants are raised to the Peerage, it is impossible to find a single reason why illustrious medical men should not be so distinguished. On the other hand, it would be easy to put forward many valid reasons why physicians and surgeons of the highest scientific attainments should take precedence of any or all of those classes.

